# Effects of Enterprise 2 stent-assisted coil embolization for wide-necked intracranial aneurysms

**DOI:** 10.1186/s41016-022-00298-w

**Published:** 2022-10-02

**Authors:** Yangyang Zhou, Qichen Peng, Shiqing Mu

**Affiliations:** Beijing Neurosurgical Institute and Department of Interventional Neuroradiology, Beijing Tiantan Hospital, Capital Medical University, Beijing, 100070 China

**Keywords:** Intracranial aneurysm, Enterprise 2 stent, Coiling, Stent malposition, Treatment outcome

## Abstract

**Background:**

This study analyzed the safety and efficacy of Enterprise 2 stent-assisted coil embolization for wide-necked intracranial aneurysms by examining stent-vessel apposition, operative complications, embolization outcomes, and clinical outcomes.

**Methods:**

We retrospectively reviewed the medical records of patients with wide-necked intracranial aneurysms who underwent Enterprise 2 stent-assisted coiling in our hospital from November 2018 to October 2019. Intraoperative VasoCT was performed immediately after stent release in a continuous cohort of patients to observe stent-vessel apposition. Patient demographic, clinical, and imaging data were recorded and analyzed.

**Results:**

A total of 106 wide-necked aneurysms in 106 patients were treated. Stent release was successful in all patients. Twenty-one patients were enrolled consecutively for VasoCT scanning, and incomplete stent apposition was observed in 5 (23.8%). Perioperative complications occurred in 10 patients (9.4%): cerebral infarction in 6, intraoperative coil prolapse in 1, puncture site pseudoaneurysm in 1, deep vein thrombosis at multiple sites in 1, and transient brainstem mass effect in 1. Among the 95 aneurysms with angiographic follow-up, embolization was satisfactory (Raymond–Roy classifications I and II) in 89 (93.7%). Hyperlipidemia was an independent risk factor for incomplete aneurysm occlusion. At the last clinical follow-up, seven patients had a poor clinical outcome (modified Rankin Scale score ≥ 3). Independent risk factors for poor outcomes were preoperative subarachnoid hemorrhage at presentation and cerebral infarction.

**Conclusion:**

Enterprise 2 stent-assisted coiling for treatment of wide-necked intracranial aneurysms showed good safety and efficacy; however, incomplete stent apposition can still occur in vessels with a large curvature. Preoperative subarachnoid hemorrhage at presentation and cerebral infarction are the main reasons for poor clinical outcomes after stent-assisted coil embolization.

## Background

Intracranial aneurysm rupture is the most common cause of subarachnoid hemorrhage (SAH) and is associated with high rates of disability and mortality [1]. Early aneurysm treatment is usually indicated after detection. Endovascular therapy is currently the preferred method. Stent-assisted coil embolization has advantages over simple embolization when treating wide-necked or other refractory aneurysms [2]. Stent use can prevent herniation of coils into the parent artery and promote repair of the aneurysmal neck, which can prevent aneurysm growth and reduce the incidence of recanalization [3]. The Enterprise Vascular Reconstruction Device (EP) (Codman Neurovascular, Miami Lakes, FL, USA) is characterized by a self-expandable, closed-cell design and has been widely used to treat wide-necked aneurysms. Its safety and efficacy have been demonstrated in numerous studies [4–6]. The second-generation product, the Enterprise 2 Vascular Reconstruction Device (EP2), features upgrades including increased maximum diameter (5.0 mm) and improved stent strut geometry (increased amplitude of the sine wave wire structure). These upgrades aim to reduce the incidence of operative complications by achieving better stent–vessel wall apposition [7]. The implantable stent is made of nitinol with a closed mesh design. The stent has four markers on each end and is covered by a layer of polymer. The transfer guide wire consists of a nitinol guide wire core with an opaque ray mark. The inductor consists of a polymer tube with a tapered distal end. It is designed to prevent stent damage and to create a continuous passage for the stent from the applicator to the perfusion catheter. EP2 stent is usually used with a 0.021 “inner diameter and 5-cm distal perfusion catheter.” This study examined the safety and efficacy of EP2 stent-assisted coil embolization of wide-necked aneurysms in a real-world clinical setting.

## Methods

### Patient selection

We retrospectively reviewed the medical records of all patients with wide-necked intracranial aneurysms who were treated with EP2 stent-assisted coil embolization from November 2018 to October 2019 in Beijing Tiantan Hospital. Wide-necked aneurysms were defined as those with an aneurysm neck > 4 mm in diameter or a neck diameter < 4 mm with dome-to-neck ratio < 2. Exclusion criteria were as follows: (1) simultaneous treatment of another cerebrovascular disease such as arteriovenous malformation, (2) modified Rankin Scale (mRS) score ≥ 3, (3) contraindication to antithrombotic or anticoagulant therapy, (4) history of serious adverse reaction to contrast agent, (5) pregnancy or lactation, (6) contraindication to endovascular treatment on angiography such as severe vascular stenosis, and (7) concurrent liver disease, kidney disease, congestive heart failure, or malignancy. Institutional review board approval was obtained. Institutional review board approval was obtained. All patients provided written informed consent.

### Data collection and follow-up

Patient demographic and clinical data, including gender, age, neurologic condition, lifestyle, and underlying diseases, were recorded. Imaging data were examined to determine aneurysm number, size, shape, and parent artery. Operative details and complications were recorded. Aneurysm condition was determined immediately after the operation and at imaging follow-up by two experienced neurointerventional physicians. The Raymond–Roy classification (RRC) system was used to categorize aneurysm embolization: class I, complete obliteration; class II, residual neck; and class III, residual aneurysm. Class I and class II were considered satisfactory imaging outcomes. Imaging follow-up modalities included digital subtraction angiography (DSA), computed tomography angiography (CTA), and magnetic resonance angiography (MRA). The first imaging follow-up was conducted 3 to 6 months postoperatively. If the patient had satisfactory findings and no obvious neurological symptoms, the second follow-up was conducted 1 to 2 years later. Patient symptoms were obtained from the medical record and via telephone. Clinical outcome was assessed using the modified Rankin Scale (mRS) score. Good outcome was defined as mRS score 0–2; poor outcome was defined as mRS score 3–6.

### Endovascular procedure

All patients with unruptured aneurysms were required to take oral aspirin 100 mg per day and clopidogrel 75 mg per day for at least 5 days before the operation. For patients with ruptured aneurysms requiring emergency treatment, single doses of aspirin 300 mg and clopidogrel 300 mg were administered before the operation. Patient reactivity to these two antiplatelet drugs was routinely tested; if a patient had a low response to clopidogrel, it was replaced with ticagrelor. All operations were performed under general anesthesia. Intravenous heparin 3000 IU was administered immediately after induction of general anesthesia, followed by 1000 IU every hour. After unilateral femoral artery puncture was performed using the modified Seldinger technique, a 6 Fr arterial sheath was placed. Angiography of the intracranial arteries was performed to identify the aneurysm and parent artery. A stent microcatheter (Prowler Plus Select; Codman Neurovascular) was placed in the appropriate position at the distal end of the parent artery under microguide wire guidance. The coil microcatheter (Echelon 10; Covidien, Irvine, CA, USA) was then advanced into the aneurysm. After the first coils were released through the microcatheter and the coil basket was well formed, the stent was completely released. The aneurysm was then filled with coils. In patients who underwent intraoperative VasoCT (high-resolution contrast-enhanced cone-beam computed tomography), the stent was completely released before coil packing. Coiling was completed once intraoperative imaging indicated complete aneurysm embolization. After the operation, all patients received aspirin 100 mg per day for 1 year and clopidogrel 75 mg per day for 3 months.

### VasoCT technology

VasoCT creates three-dimensional volumetric images using rotation of a C-arm. Scanning was carried out on an AlluraXper FD20 system (Philips, Amsterdam, Netherlands), and the contrast agent (50 mL:33.9 g) was diluted to 7–10%. The injection rate was adjusted to 3 mL/s with an injection volume of 60 mL. The injection pressure was set to 300 psi, and ray delay was set to 2 s. Imaging parameter settings were as follows: C-arm rotation time, 20 s; imaging frames, 620; rotation angle, 220°; rotational speed, 10°/s; maximum field of vision, 22 cm × 22 cm; voltage, 80 kV; and radiation dose, 49 mGy. Stent and vessel images were reconstructed on a 3D workstation. The obtained volume image was optimized for stent reconstruction with a resolution of 512^3^. Maximum density projection mode was selected, and contrast and layer width were adjusted to create the clearest image.

### Statistical analysis

Statistical analysis was conducted using SPSS software version 24.0 (IBM Corp., Armonk, NY, USA). Continuous variables are expressed as means with standard deviation. Categorical variables are expressed as frequencies. Data were compared between groups using the independent-samples *t* test, chi-square test, or Fisher’s test as appropriate. Univariate analysis was performed to identify risk factors for incomplete aneurysm occlusion and poor clinical outcome. Multivariate logistic regression analysis was performed using univariate variables with *P* < 0.2. *P* < 0.05 was considered significant.

## Results

In total, 106 wide-necked aneurysms in 106 patients were treated with EP2 stent-assisted coil embolization. Table 1 shows the patient and aneurysm characteristics overall and according to presentation with aneurysmal SAH. Nineteen patients had a ruptured aneurysm with SAH. Patient and aneurysmal characteristics did not significantly differ between patients with ruptured and unruptured aneurysms.

**Table 1:** Patient and aneurysm characteristics

Characteristic	Total	nSAH	SAH	*P* value
Number of patients	106	87	19	
Male sex, *n* (%)	40 (37.7)	33 (37.9)	7 (36.8)	0.796
Age (years), mean ± SD	56.3 ± 10.9	55.5 ± 11.1	59.8 ± 9.2	0.119
Smoking, *n* (%)	17 (16.0)	14 (16.1)	3 (15.8)	< 0.999
Alcohol use, *n* (%)	15 (14.2)	12 (13.8)	3 (15.8)	0.730
Hypertension, *n* (%)	49 (46.2)	41 (47.1)	8 (42.1)	0.802
Hyperlipidemia,* n* (%)	11 (10.4)	8 (9.2)	3 (15.8)	0.411
Diabetes, *n* (%)	8 (7.5)	8 (9.2)	0	0.346
Cerebral ischemic comorbidities, *n* (%)	24 (22.6)	23 (26.4)	1 (5.3)	0.554
Aneurysm location				
Carotid artery	59 (55.7)	49 (56.3)	10 (52.6)	0.731
Distal Circle of Willis^a^	33 (31.1)	28 (32.2)	5 (26.6)	0.430
Vertebrobasilar system, *n* (%)	14 (13.2)	10 (11.5)	4 (21.1)	0.556
Angle of parent vessels (degree)	112.2 ± 31.6	113.5 ± 30.8	106.1 ± 29.9	0.343
Diameter of parent vessel (mm)	3.5 ± 1.2	3.6 ± 1.2	3.2 ± 1.1	0.202
Maximum diameter of, mm, mean ± SD	6.8 ± 3.6	6.9 ± 3.7	6.1 ± 2.4	0.351
Neck width (mm), mean ± SD	4.8 ± 2.6	4.9 ± 2.6	4.8 ± 2.2	0.895

*SAH* Subarachnoid hemorrhage, *nSAH* No subarachnoid hemorrhage, *SD* Standard deviation; ^a^Distal Circle of Willis includes the middle cerebral artery, anterior cerebral artery, anterior communicating artery, and posterior communicating artery

### Stent placement and stent-vessel apposition

**Fig. 1:**
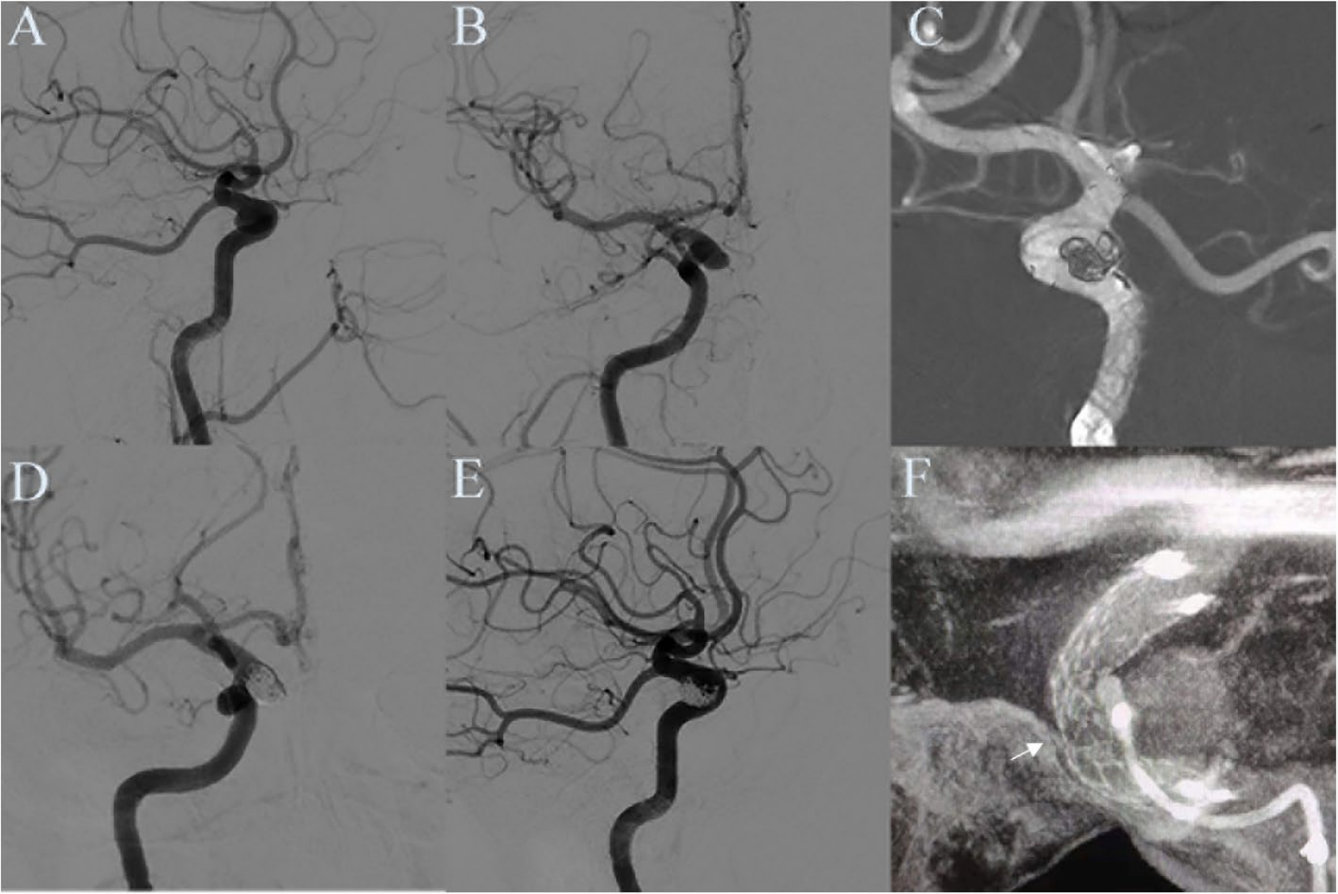
Stent body malposition.** A**,** B** Digital subtraction angiography shows a posterior communicating artery aneurysm in a 50-year-old man. **C** Stent-assisted coiling was performed using a 4.0 × 23 mm EP2 stent. **D**, **E** Angiography immediately after the operation showed near-complete aneurysm embolization. **F** VasoCT showed incomplete stent apposition at the stent body (white arrow)

**Fig. 2:**
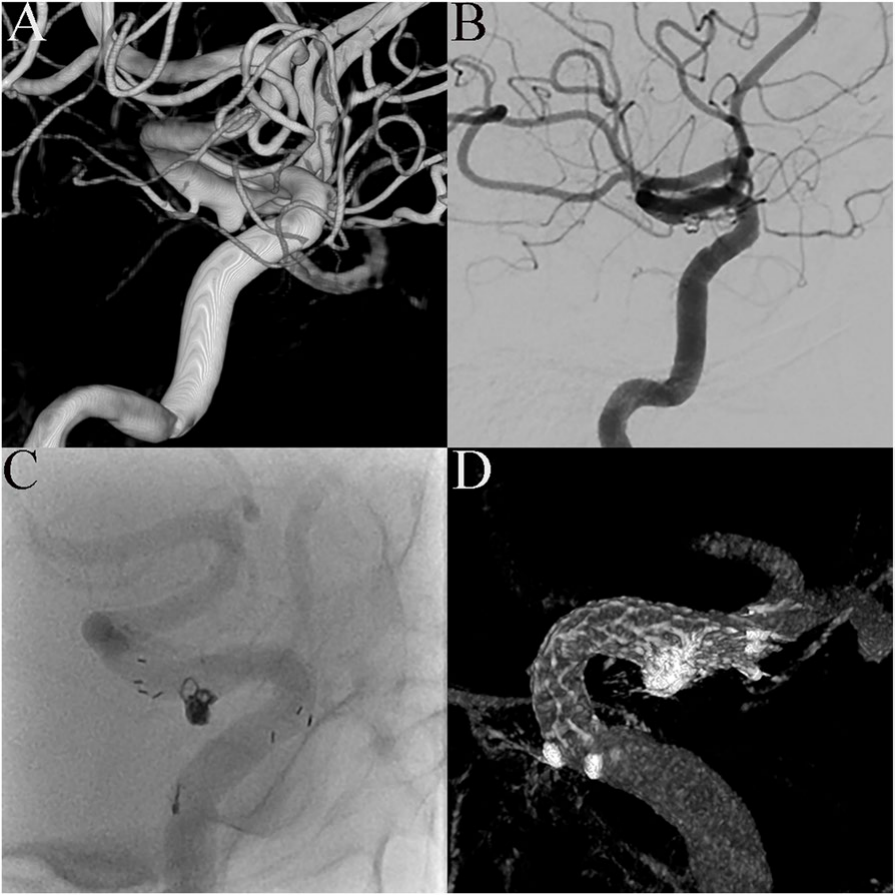
Stent opening malposition.** A** Digital subtraction angiography shows an aneurysm of the communicating segment of the left internal carotid artery in a 45-year-old man. **B** Stent-assisted coiling with a 4.0 × 23 mm EP2 stent was performed. Angiography performed immediately after the operation showed complete aneurysm embolization. **C** Disordered distribution of proximal stent markers was observed under fluoroscopy. **D** VasoCT showed malposition at the proximal opening of the stent

One hundred six stents were successfully implanted in 106 patients. All stents were successfully delivered to the aneurysm site and deployed for a technical success rate of 100%. To assess EP2 stent opposition in the tortuous vessels, we selected aneurysms within the internal carotid artery from the cavernous to the ophthalmic segment. VasoCT was performed in 21 consecutive patients: a total of five stents showed malposition, two at the stent body (Fig. 1) and three at the end opening (Fig. 2).

### Aneurysm embolization status

The frequency of satisfactory imaging outcomes (RRC class I/II) immediately after embolization in the unruptured and ruptured aneurysm groups was 87.4% and 89.5%, respectively (*p* < 0.999). A total of 95 (89.6%) patients received angiographic follow-up. The mean follow-up was 9.4 months. The frequency of satisfactory outcomes at follow-up was 93.8% and 93.3%, respectively. No significant differences were found between the two groups. Detailed imaging follow-up results of the two groups are shown in Table 2. The independent risk factors for incomplete aneurysm embolization were incomplete embolization of the aneurysm immediately postoperatively (odds ratio [OR] 63.61; 95% confidence interval [CI], 6.284–643.852; *P* < 0.001) and hyperlipidemia (OR 7.931; 95% CI, 1.346–46.737; *P* = 0.022) (Table 3).

**Table 2:** Treatment outcomes and follow-up

Result	Total	nSAH group	SAH group	*P* value
Number of aneurysms	106	87 (82.1)	19 (17.9)	
Operative time (minutes) mean ± SD	126.1 ± 51.5	121.9 ± 46.2	145.5 ± 69.0	0.168
Postoperation immediate aneurysm occlusion, RRC, *n* (%)				
I + II	93 (87.8)	76 (87.4)	17 (89.5)	< 0.999
Ninty-five aneurysms with angiographic follow-up, RRC, *n* (%)				
I + II	89 (93.7)	75 (93.8)	14 (93.3)	< 0.999
Imaging follow-up time (months)	9.4 ± 4.6	9.8 ± 4.7	7.8 ± 3.9	0.087
Imaging follow-up methods				
DSA	36 (37.9)	28 (32.2)	8 (42.1)	
CTA	57 (53.8)	46 (52.9)	11 (57.8)	
MRA	2 (2.2%)	2 (2.3)	0	
mRS = 0–2	99 (93.4)	83 (95.4)	16 (84.2)	0.107
Clinical follow-up time (months)	19.7 ± 2.3	19.8 ± 2.3	19.3 ± 2.3	0.438
Procedure-related complications, *n* (%)	10 (9.4)	7 (8.0)	3 (15.8)	0.380

*SAH* Subarachnoid hemorrhage, *nSAH* No subarachnoid hemorrhage, *SD* Standard deviation, *RRC* Raymond–Roy classification, *mRS* Modified Rankin scale, *DSA* Digital subtraction angiography, *CTA* Computed tomography angiography, *MRA* Magnetic resonance angiography

**Table 3:** Risk factors of incompletely occluded

Variables	Univariate analysis			Multivariate logical regression analysis		
	*P*	OR	95%CI	*P*	OR	95%CI
Hyperlipidemia	0.025	8.889	1.557–50.763	0.022	7.931	1.346–46.737
Vertebrobasilar system	0.189	3.545	0.580–21.685	0.309	2.736	0.394–19.006
Immediate aneurysm occlusion, RRC III	< 0.001	69.167	6.925–690.823	< 0.001	63.610	6.284–643.852

*RRC* Raymond–Roy classification, *OR* Odds ratio, *CI* Confidence interval

### Operative complications

**Fig. 3:**
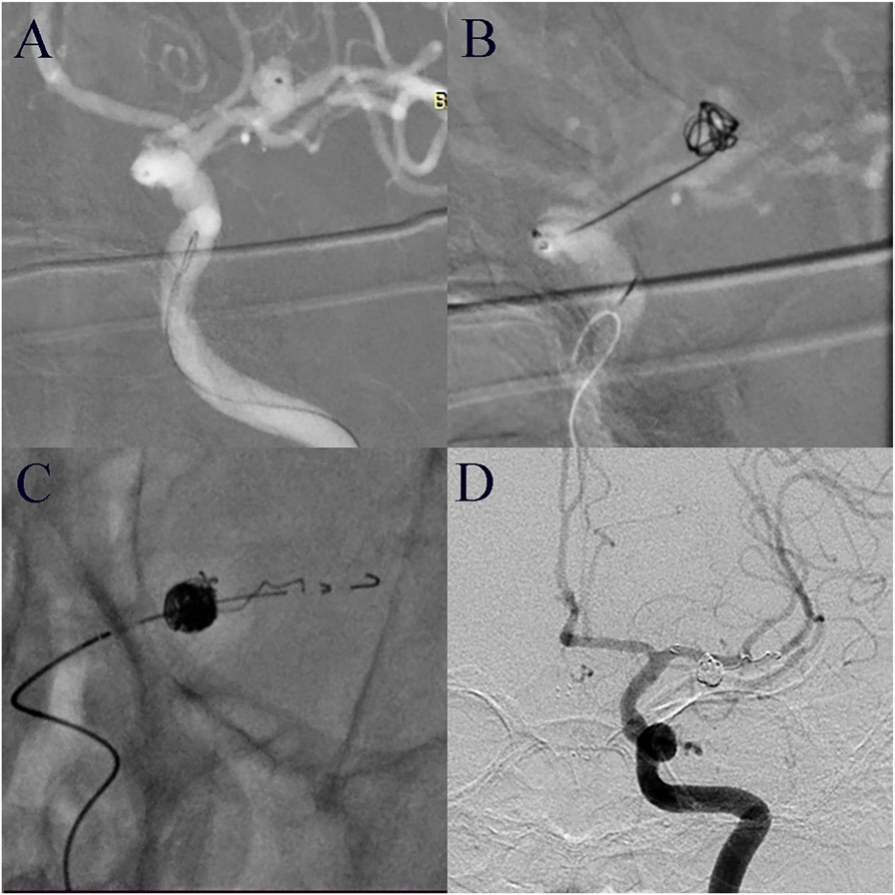
Salvage after coil prolapse. **A** Digital subtraction angiography shows a left middle cerebral artery aneurysm in a 56-year-old man. **B** This aneurysm ruptured during anesthesia induction and was treated with emergency coil embolization. **C** The last coil prolapsed into the parent artery, and therefore, an EP2 stent was placed. **D** Angiography immediately after the operation showed complete aneurysm embolization

**Fig. 4:**
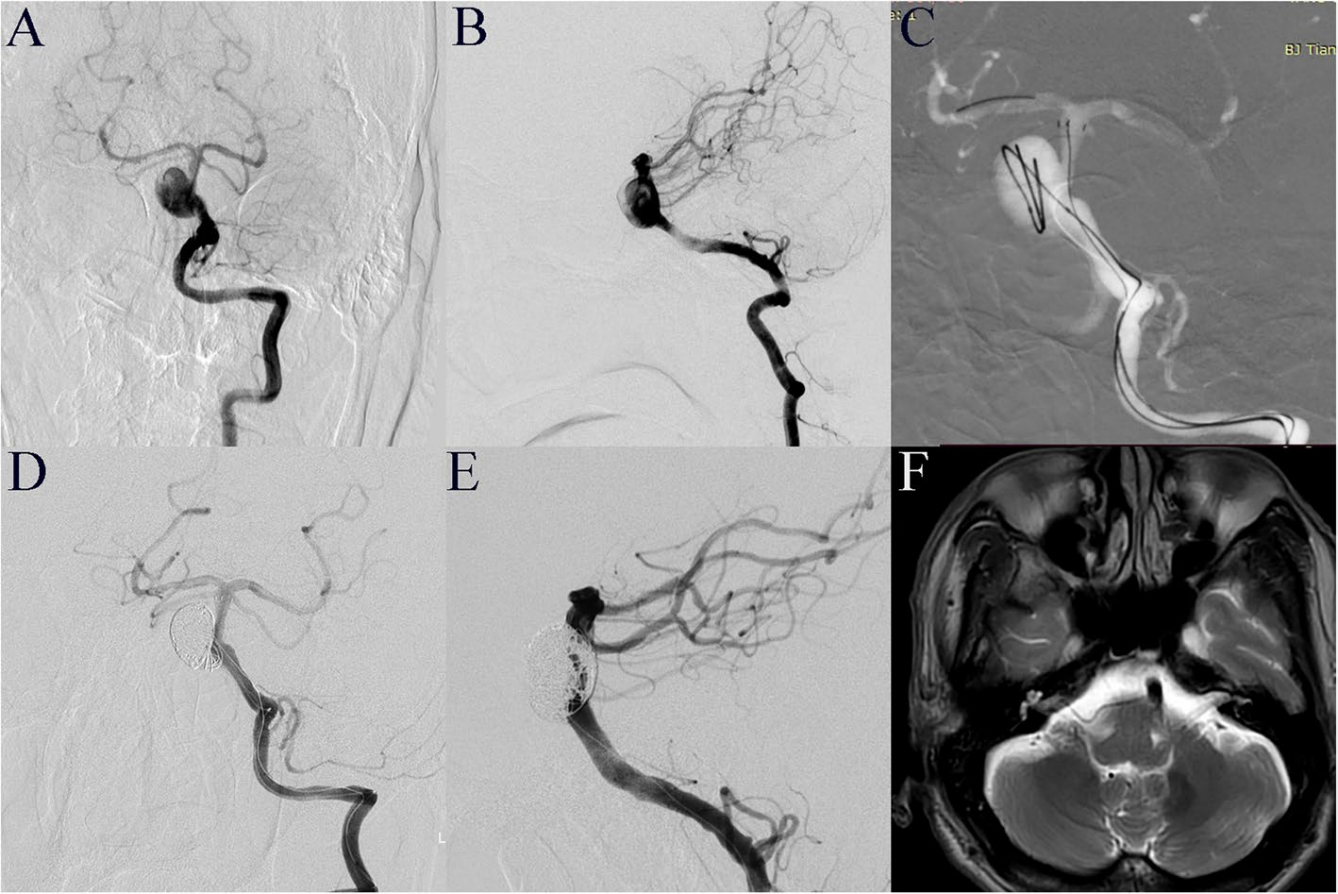
Infarction.** A**, **B** Left vertebral artery angiography demonstrates a basilar artery aneurysm in a 56-year-old man. **C** EP2 stent-assisted coiling was performed. **D** Angiography immediately after the operation showed complete aneurysm embolization and patency of the parent artery. **F** Magnetic resonance imaging obtained because of right upper arm weakness on the second day after the operation showed new infarction

One patient experienced aneurysmal rupture during anesthesia induction and underwent rapid simple coil embolization; when the microcatheter was withdrawn, the last coil prolapsed into the parent artery, and therefore, an EP2 stent was placed (Fig. 3). Six patients developed cerebral infarction within the first 3 days of the operation. Infarction presented with varying degrees of the limb muscle weakness or speech impairment and was confirmed by computed tomography (CT) or magnetic resonance imaging (Fig. 4). One patient experienced transient brain stem mass effect after the operation and presented with binocular diplopia and left gaze preference. Another developed a pseudoaneurysm at the femoral puncture site that ruptured and required repair; this patient recovered well. Deep vein thrombosis at multiple sites occurred in one patient with pre-existing atrial fibrillation. Complications did not significantly differ between the unruptured and ruptured aneurysm groups.

### Clinical outcome

The study cohort comprised 106 patients; 42 patients had no neurological symptoms on admission (mRS score = 0), 55 were symptomatic but had no disabilities (mRS score = 1), and nine had mild disabilities (mRS score = 2). All patients underwent clinical follow-up (mean 19.7 months). No deaths occurred. Seven patients experienced poor clinical outcomes (mRS score ≥ 3). Among these seven patients, two had aneurysms as unexpected findings and postoperative infarct complications (final mRS scores 3 and 4, respectively); three patients with ruptured aneurysms had neurological deficits postoperatively (final mRS scores 3, 4, and 5, respectively); two patients had cerebral infarction before admission, manifested by decreased limb muscle strength (final mRS scores 3 and 4, respectively). Although the unruptured aneurysm group had fewer complications and better clinical outcomes than the ruptured aneurysm group, univariate analysis showed that these intergroup differences were not significant. The final multivariate analysis showed that the independent risk factors for poor clinical outcome were preoperative SAH (OR 20.992; 95% CI, 1.601–275.282; *P* = 0.020) and cerebral ischemic comorbidity (OR 15.100; 95% CI, 1.414–161.297; *P* = 0.025) (Table 4).

**Table 4:** Risk factors of poor clinical outcome

Variables	Univariate analysis			Multivariate logical regression analysis		
	*P*	OR	95%CI	*P*	OR	95%CI
SAH	0.107	3.891	0.794–19.073	0.020	20.992	1.601–275.282
Diabete	0.088	6.133	0.978–38.466	0.068	10.349	0.842–127.273
Cerebral ischemic comorbidities	0.038	5.614	1.158–27.210	0.025	15.10	1.414–161.297
Multiple aneurysms, *n* (%)	0.138	3.375	0.694–16.413	0.409	2.166	0.346–13.561

*SAH* Subarachnoid hemorrhage, *RRC* Raymond–Roy classification, *OR* Odds ratio, *CI* Confidence interval

## Discussion

The goal of intracranial aneurysm treatment is rupture prevention because rupture is associated with high rates of disability and mortality [8]. Treatment options include craniotomy for surgical clipping and endovascular intervention. Compared with craniotomy, endovascular therapy is less invasive and associated with lower risk and faster recovery. The International Subarachnoid Aneurysm Trial demonstrated the superiority of endovascular treatment over surgical clipping [9], which has resulted in endovascular therapy becoming the primary treatment method [10]. However, simple coil embolization of wide-necked aneurysms is challenging. Stent-assisted coiling can overcome the challenge by preventing coil prolapse and allowing dense coil packing. In addition, the stent can serve as a blood flow diverter and a scaffold to promote attachment of endothelial cells in the aneurysmal neck, which can reduce the incidence of aneurysm recanalization [3, 11].

The EP stent has been widely used around the world in stent-assisted coiling procedures. Compared with the EP stent, the EP2 stent has improved stent compliance and marker visibility; the transmission device also has an additional flushing port to simplify flushing. Furthermore, the expansion diameter and the amplitude of the sine wave in its geometric structure are greater. Although both stents are composed of a sine wave geometric structure, the increased sine wave amplitude of the EP2 stent makes it more adaptable to the physical characteristics of the vessel curve. Therefore, the stent can be elongated at the outer arc of the curve and compressed at the inner arc, which makes the EP2 stent more suitable for curved parent arteries. Heller et al. [12] first reported that EP stents were prone to malposition in vessels with a large curvature. In 2016, Kono et al. [13] tested release of the EP and EP2 stents in vitro and found that the gap between the stent and the tube wall was twice as large for the EP stent, indicating that the EP2 stent has better stent-vessel apposition. Herweh et al. [7] first studied the application of the EP2 stent in clinical patients and reported good safety and effectiveness; however, incomplete stent apposition still occurred in the vessels with a large curvature. Kato et al. [14] used the double volume fusion technique based on C-arm CT to compare stent-vessel apposition of three stents (EP2, Neuroform, and Atlas) and found the frequency of crescent sign detection in the bending vessels was highest for the EP2 stent (27%, 8%, and 0, respectively). Chen et al. [15] used VasoCT to observe EP2 stent release and reported that 6 of 25 stents (24%) showed malposition: 2 showed kinking and the other 4 showed large gaps between the stent and vessel wall. In our study, VasoCT was performed on 21 stents that were all implanted in the vessels with large curves, 5 (23.8%) showed incomplete stent apposition, two at the stent body, and three at the end opening. Therefore, the EP2 stent still may exhibit incomplete stent apposition in the vessels with a large curvature. Our study showed that the stent placement is less satisfactory in a real-world clinical setting than in in vitro experiments. The complex physiological bending of the blood vessels encountered in vivo may be an explanation. However, we did not find an association between incomplete stent apposition and postoperative infarction. Further studies are warranted to explore the relationship between the two.

In a multicenter study of 141 patients with 143 intracranial aneurysms treated with EP stent-assisted coiling, Mocco et al. [6] reported that 76% of aneurysms eventually achieved RRC class I or II outcomes with temporary and permanent complication rates of 6% and 2.8%, respectively, and a 2% mortality. Another study of EP-stent assisted coiling by Mocco et al. [16] reported a 3% rate of stent-related stenosis or occlusion. A meta-analysis of 1007 aneurysms in 915 patients treated with the EP stent reported incidences of thrombotic events and intraoperative rupture of 4.9% and 1.2%, respectively, with satisfactory embolization results in 85.45% of aneurysms [5]. In our study, 93.7% of aneurysms were finally completely or nearly completely embolized, which was better than the results reported in the abovementioned previous studies, indicating that the modified structure made the EP2 stent superior to the EP stent. In addition, we found that hyperlipidemia was an independent risk factor for incomplete aneurysm embolization. Hyperlipidemia is an important factor in atherosclerosis in which endothelial cells are damaged, and this process may also exist in patients with intracranial aneurysm. Therefore, hyperlipidemia may affect the endothelialization of intracranial aneurysm, thus hindering complete aneurysm occlusion. No aneurysm recanalized and angiographic follow-up showed stenosis of the parent artery in only one patient. It is likely that the structural upgrade and increased stent diameter of the EP2 stent allowed dense embolization of the aneurysm and contributed to these results. In our study, the perioperative cerebral infarction rate was 5.7%, and permanent complication and mortality rates were 1.9% and 0%, respectively, which were comparable to the rates reported in the abovementioned previous studies. Six patients developed infarction within 3 days postoperatively, which was the primary factor affecting clinical outcome in patients with unruptured aneurysm. However, we were unable to determine the exact cause of infarction (delayed thrombosis, cerebral vasospasm, or other). Multivariate analysis showed that preoperative SAH and cerebral infarction were independent risk factors for poor clinical outcome. The presence of concomitant cerebral infarction usually means that the patient has concomitant chronic diseases (such as diabetes and hypertension), and the presence of cerebrovascular stenosis may be the direct cause of cerebral infarction. Given that cerebral infarction results in neurological deficits, patients with cerebral infarction are more likely to develop new ischemic events after stent-assisted coil therapy and are thus more likely to have poor outcomes. Similarly, as SAH caused by aneurysm rupture often results in irreversible motor or sensory impairment caused by brain tissue damage, patients with SAH are more likely to have poor postoperative outcomes. These results suggest that the routine cerebrovascular examination to prevent aneurysm rupture and cerebral infarction remains the key to improving clinical outcome.

On the basis of our experience with EP2 stent-assisted coiling, several stent characteristics and technical details should be discussed. The EP2 stent delivery device adds a unique headless end design available for selection. This design can avoid causing vascular endothelial injury, branch injury, and vascular spasm in distal tortuous vessels, vessels with smaller diameter, and vessels with a large number of branches. After stent release, the microcatheter can be retracted without causing stent displacement. In addition, compared with the EP stent, the EP2 stent markers are more radiopaque and can be visualized more clearly. Although the EP2 stent has an upgraded structure to improve its attachment, the structure of the closed-cell stent itself can result in incomplete stent apposition, which can be minimized using specific release techniques. Chihara et al. [17] conducted an EP2 stent release test in vitro and confirmed that the “release along the outer arc of the vessel” technique can effectively improve the stent-vessel apposition compared with the “Heller push–pull technique.” The “Heller push–pull technique” involves pulling the stent microcatheter to make the stent adhere to the internal arc of the curved vessel when the stent is released, and adhering the stent to the external arc of the curved vessel when the guide wire is pushed; these steps are repeated until the stent is completely released. The “releasing along the outer arc of the vessel” technique involves keeping the stent released along the outer arc of the vessel: first, the marks are symmetrically covered at the aneurysm neck, and appropriate forward force is slightly applied to the transmission guidewire. At the same time, the microcatheter is pulled back to slowly release the stent. After 5–10 mm of stent release, the force on the microcatheter and transmission guide wire is balanced to stabilize the position of the stent in the initial release stage. Then, during the whole release process, the delivery guidewire is kept with appropriate thrust, so that the stent is attached to the outer edge of the curved vessel.

### Study limitations

Our study has several limitations. First, it was a single-center retrospective study with a small sample size. Second, some patients were lost to follow-up after discharge and some with suboptimal imaging data were excluded, and thus, there may be some deviation between the final research results and the actual results. Third, VasoCT was performed in a small number of patients. Further studies are warranted to confirm our results.

## Conclusion

The study demonstrated that EP 2 stent-assisted coiling of wide-necked intracranial aneurysms is a safe and effective treatment; however, incomplete stent apposition can still occur in the vessels with a large curvature. Preoperative SAH and cerebral infarction are the main reasons for poor clinical outcome after stent-assisted coil embolization.
